# Reference values for exhaled nitric oxide (reveno) study

**DOI:** 10.1186/1465-9921-7-94

**Published:** 2006-06-30

**Authors:** Mario Olivieri, Giorgio Talamini, Massimo Corradi, Luigi Perbellini, Antonio Mutti, Claudio Tantucci, Mario Malerba

**Affiliations:** 1 Department of Medicine and Public Health, University of Verona, Italy; 2 Department of Clinical Medicine, Nephrology and Health Sciences, University of Parma, Italy; 3 Department of Internal Medicine, University of Brescia, Italy

## Abstract

**Background:**

Despite the widespread use of fractional exhaled nitric oxide (FE_NO_) as a biomarker of airways inflammation, there are no published papers describing normal FE_NO _values in a large group of healthy adults.

**Objective:**

The aim of this study was to establish adult FE_NO _reference values according to the international guidelines.

**Methods:**

FE_NO _was measured in 204 healthy, non-smoking adults with normal spirometry values using the on-line single-breath technique, and the results were analysed chemiluminescently.

**Results:**

The main result of the study was the significant difference in FE_NO _values between men and women, thus indicating that gender-based reference FE_NO _values are necessary. The FE_NO _levels obtained at expiratory flows of 50 ml/s ranged from 2.6 to 28.8 ppb in men, and from 1.6 to 21.5 ppb in women.

**Conclusion:**

We propose reference FE_NO _values for healthy adult men and women that could be used for clinical and research purposes.

## Background

The presence of nitric oxide (NO) in exhaled air was first described in 1991 by Gustafsson *et al*.[1], and this was soon followed by a number of publications reporting high fractional concentrations of orally exhaled NO (FE_NO_) in subjects with various pulmonary diseases [2]. FE_NO _is generally measured on line by having the subject blow directly into the analyser and obtaining immediate results [3], but breath can also be collected remotely into inert bags and analysed subsequently (off line) [3].

Although the pathophysiological meaning is still unclear [4], it has been demonstrated that NO levels in exhaled air are higher in asthmatics than in healthy subjects, increase during spontaneous or induced asthma exacerbations, and decrease after anti-inflammatory treatment [5].

Many studies have clearly demonstrated that a number of factors can affect FE_NO _values, and so the European Respiratory Society (ERS) and American Thoracic Society (ATS) established particular recommendations for exhaled and nasal NO measurements in 2005 [6] in order to allow the comparison of data from different research centres.

Clinicians and researchers seeking to apply FE_NO _measurements in everyday practice are obviously interested in knowing what are normal FE_NO _values in healthy subjects, but very few attempts have been made to establish such reference values, and experimental findings are usually only compared with those observed in the healthy controls recruited for any particular study. Buchvald *et al*. [7] have recently found that upper normal FE_NO _levels in children aged 4–17 years ranged from 15 parts per billion (ppb) in the youngest to 25 ppb in adolescents, with a mean increase of 1 ppb per year. To the best of our knowledge, there are no published studies indicating similar reference values for adults.

The aim of this study was to establish reference adult FE_NO _values according to the international guidelines.

## Materials and methods

### Study subjects and protocol

This open-label study was conducted in three Italian centres (Brescia, Parma and Verona) and recruited local medical school students and colleagues, who were given a short description of the project, and the inclusion and exclusion criteria.

Healthy subjects were defined as individuals with normal spirometry values and without a history of any significant diseases. Furthermore, in accordance with the ATS/ERS guidelines [6], particular care was taken to avoid the known confounding factors that may affect FE_NO _measurements: in particular, smokers and ex-smokers were excluded; none of the volunteers was taking any drug or medication or had experienced a recent upper or lower airways infection, and none reported any clinical manifestation of allergic diseases or positive skin prick tests for common inhalant allergens.

The study was approved by the Ethics Committee of each centre and all of the participants gave their written informed consent.

### Fractional exhaled NO measurements

Fractional exhaled NO (FE_NO_) was measured using a chemiluminescence analyser (CLD88, Ecomedics, Switzerland) whose lower and upper limit of detection (LOD) was respectively 0.06 ppb and 100 ppb. The same type of instrument was used at all of the centres, and was calibrated at 0 and 100 ppb as recommended by the manufacturer.

FE_NO _was measured in accordance with international guidelines [6]. Briefly, after inhaling to total lung capacity, the subjects exhaled through a mouthpiece equipped with a 0.2-μm pore size bacterial filter into an exhalation circuit consisting of an ultrasonic flow meter, one-way valve and one sampling port. NO was sampled directly in the analyser (at a flow rate of 250 ml/min) through a Teflon side arm tube attached to the sampling port. The sampling tube was 60 cm long with an internal diameter of 1/8 of in. Both expiratory flow and FE_NO _values were simultaneously displayed on a computer attached to the analyser. FE_NO _was measured before the subjects underwent spirometry.

Different expiratory flow rates were ensured by placing expiratory resistors (Breath kit, Sievers Instruments, USA) in the exhalation circuit, which yielded expiratory flow rates of 50, 100 and 200 ml/s. The subjects were asked to exhale at a constant flow, which they could readily see displayed on the computer screen in the form of a bar that remained red until target flow was obtained, and then turned green; if the flow dropped below or increased above the desired range, the green bar changed back to red. Although the target expiratory flows were strictly controlled and maintained during the expiration, a tolerance of ± 10% was considered acceptable, and the exhalation continued until a stable plateau had been reached.

Three FE_NO _plateau measurements varying by <10% were made at each flow rate, and the average value was recorded. As the subjects inhaled ambient air, its NO concentration was measured at the time of each test and, if high (>30 ppb), the data were discarded. The influence of ambient NO levels was further excluded by placing an NO-scrubbing filter in the inspiratory limb of the collection apparatus. The data were stored on a computer and analysed using NO analysis software.

### Spirometry

The patients underwent spirometry using a spirometer connected to a computer for data analysis (Vmax 22, Sensor Medics, Yorba Linda, CA, USA), and FEV_1 _and FVC were measured in accordance with the ATS standard procedure [8].

### Data analyses

We first analysed the three subgroups of subjects from each centre and then all of the subjects as a whole. As there was no significant difference between the two analyses, for the sake of simplicity, we shall here describe only the results of the first.

Spearman's correlation test was used to verify the correlations between the variables. Between-group comparisons were made using non-parametric analysis of variance (Kruskal-Wallis test) and, if significant, the Mann-Whitney U test (M-W test). Logarithmic transformation was applied to the NO values in order to normalise the curve and the groups were compared using ANOVA; however, in order to simplify the reading, the data are presented as their original values and analysed non-parametrically. Bonferroni's correction for multiple tests was applied.

In the multivariate analysis of the odds ratio estimates, logistic regression was carried out backwise with pre-assigned *P *values of > 0.05 controlling step removal; the model was evaluated using three goodness-of-fit chi-square statistics.

All of the analyses were made using SPSS Rel. 13.0 statistical package (SPSS Inc., Chicago, IL).

## Results

Table 1 shows the demographic data, physical and spirometric parameters, and FE_NO _values. The demographic data, physical parameters, and mean spirometric and FE_NO _values of the healthy non-smoking subjects studied in the three centres were pooled as there were no significant between-centre differences (data not shown).

**Table 1:** Demographic data, physical parameters and FE_NO _values in studied subjects. Mean values and standard deviation (SD).

	Males		Females		Total		Values		P value*
	No.	Value	No.	Value	No.	Value	Min	Max	
Age (yrs)	102	37.0 ± 9.5	102	35.0 ± 10.1	204	36.1 ± 9,9	19	59	n.s.
Weight (kg)	102	77.6 ± 12.2	102	59.9 ± 9.8	204	68.8 ± 14.2	44	112	0.001
Height (cm)	102	176	102	164	204	170	148	190	0.001
BMI (m/kg^2^)	102	25.1	102	22.2	204	23.7	17.2	35.4	0.001
BSA (m^2^)	102	1.7	102	1.5	204	1.6	1.3	2.1	0.001
FE_NO _50 (ppb)	102	11.7 ± 5.0	102	9.9 ± 4.3	204	10.8 ± 4.7	0.7	28.8	0.01
FE_NO _100 (ppb)	92	7.1 ± 3.0	86	5.6 ± 2.5	178	6.4 ± 2.9	1.7	16.9	0.001
FE_NO _200 (ppb)	93	4.4 ± 2.0	86	3.5 ± 1.4	179	4.0 ± 1.8	0.9	10.7	0.001
FVC (litres)	102	5.2 ± 0.8	102	3.8 ± 0.5	204	4.5 ± 1.0	2.3	7.1	0.001
FVC % predicted	102	108.3 ± 12.7	102	109.8 ± 12.3	204	109.1 ± 12.5	79	147.4	n.s.
FEV_1 _(litres/1 sec)	102	4.2 ± 0.6	102	3.2 ± 0.4	204	3.7 ± 0.7	2.0	5.8	0.001
FEV_1 _% predicted	102	105.7 ± 11.5	102	107.3 ± 9.3	204	106.5 ± 10.5	78	133.7	n.s.

Abbreviations: n.s. = not significant; FENO 50 = fractional exhaled nitric oxide in parts per billion (ppb) at an expiratory flow of 50 mL/sec; FENO 100 = fractional exhaled nitric oxide in parts per billion (ppb) at an expiratory flow of 100 mL/sec; FENO 200 = fractional exhaled nitric oxide in parts per billion (ppb) at an expiratory flow of 200 mL/sec; FEV1 = forced expiratory volume (litres) in one second; FVC = forced vital capacity (litres); BMI = body mass index; BSA= body surface area; Min = minimum value; Max = maximum value

* Mann-Whitney U-test comparing male and female subjects

Table 2 shows the distribution of the FE_NO _values. Of the 204 recruited subjects (male/female ratio = 1), 78 (38%) were aged 19–30, 65 (32%) were aged 31–40, 39 (19%) were aged 41–50, and 22 (11%) were aged 51–60 years. There was no significant difference in age between the sexes.

**Table 2:** Data distribution of fractional exhaled nitric oxide values.

FE_NO _ppb percentile		Total cases	5^th^	10^th^	20^th^	25^th^	30^th^	40^th^	50^th^	60^th^	70^th^	75^th^	80^th^	90^th^	95^th^
FE_NO_50	Males	102	4.5	5.5	7.3	8.6	9.0	10.3	11.4	12.5	13.8	14.4	15.1	19.2	20.6
	Females	102	3.6	4.5	5.5	6.0	7.1	8.7	9.7	10.8	12.0	13.1	13.6	16.2	18.2
	Total	204	3.8	5.0	6.2	7.3	8.2	9.4	10.4	11.7	13.1	13.7	14.5	17.3	19.7
															
FE_NO_100	Males	92	2.8	3.4	4.1	4.6	5.3	6.0	6.8	7.6	8.4	9.0	9.7	11.5	12.8
	Females	86	2.2	2.6	3.3	3.7	4.0	4.8	5.4	6.0	6.7	7.4	8.0	9.6	10.3
	Total	178	2.4	3.0	3.8	4.1	4.6	5.4	6.0	6.8	7.7	8.1	8.8	10.4	11.7
															
FE_NO_200	Males	93	1.6	2.1	2.6	2.9	3.2	3.6	4.1	4.9	5.5	5.8	6.3	6.9	8.3
	Females	86	1.6	1.8	2.2	2.4	2.6	3.1	3.3	3.7	4.0	4.1	4.6	5.5	5.9
	Total	179	1.6	1.9	2.5	2.6	2.9	3.3	3.6	4.1	4.7	5.2	5.5	6.6	7.1

Abbreviations: FE_NO_50 = fractional exhaled nitric oxide at an expiratory flow of 50 mL/sec; FE_NO_100 = fractional exhaled nitric oxide at an expiratory flow of 100 mL/sec; FE_NO_200 = fractional exhaled nitric oxide at an expiratory flow of 200 mL/sec; ppb= parts per billion

All of the subjects underwent spirometry and FE_NO _measurement at an exhaled flow of 50 mL/s, and respectively 178 (92 men and 86 women) and 179 subjects (93 men and 86 women) also had FE_NO _measured at the exhaled flows of 100 and 200 mL/s. Twenty-five of the 26 subjects who were unable to perform the FE_NO _procedure at the exhaled flow of 200 mL/s were also unable to do so at 100 mL/s.

There were significant gender-related differences in body mass index (BMI), height, weight and body surface area (BSA), forced expiratory volume at the first second (FEV_1_), and forced vital capacity (FVC), but no gender-related difference in exhaled flow values.

At all of the studied flow, FE_NO _levels were significantly lower in the women than in the men (Table 1). FE_NO _levels did not correlate with age, lung function or anthropometric values.

There was a positive correlation between the FE_NO _values at the different exhaled flows: FE_NO_50 *vs*. FE_NO_100 r = 0.82, p < 0.001; FE_NO_50 *vs*. FE_NO_200 r = 0.74, p < 0.001; FE_NO_100 *vs*. FE_NO_200 r = 0.9, p < 0.001.

FE_NO _levels were not correlated with age (r = 0.1, p = 0.21, Spearman's test) or with lung function or anthropometric values. FE_NO _levels at all studied flows were significantly lower in females than those observed in men (Table 1).

Logistic regression analysis was performed considering sex as dependent variable and centre, weight, height, age, FEV1, FVC, FE_NO_50, FE_NO_100 and FE_NO_200 as potentially predictive factors.

Weight and FVC were identified as predictive variables able to distinguish between males and females (data not reported).

## Discussion

The primary aim of this study was to measure FE_NO _in a population of healthy controls aged 19–65 years at a flow rate of 50 mL/s using the on-line single breath technique. We also analysed FE_NO _at flow rates of 100 and 200 mL/s in order to obtain their normal FE_NO _values, and compare the ability of normal adults to expire at such different flows.

The main finding was that, even after adjusting for age, height, weight, BMI and BSA, FE_NO _values were significantly lower in women at all of the studied expiratory flows, which means that different gender-related reference values need to be applied.

FE_NO _levels at the most frequently used expiratory flow rate of 50 ml/s was 2.6–28.8 ppb in men and 1.6–21.5 ppb in women. The FE_NO _levels at 50 ml/s usually reported in studies of healthy adults fall within the 10–20 ppb range [9] but, as in the case of other biological parameters, we observed some individuals with unexplained higher or lower levels despite our strict study inclusion and exclusion criteria. It may therefore be more prudent to define normal FE_NO _values in terms of percentiles, and we would suggest considering the fifth and 95^th ^percentiles (4.5–20.6 ppb for males, and 3.6–18.2 ppb for females), as references for healthy subjects, and taking further diagnostic and clinical steps in the case of subjects whose FE_NO _levels fall outside this range.

Gender-related differences in adult FE_NO _levels were first reported by Jilma *et al*.[10], who examined the concentrations of exhaled NO and plasma nitrate, and were confirmed by Tsang *et al*.[11] in a cohort of 121 healthy non-smoking subjects, and by van der Lee *et al*.[12] However, our data were collected in accordance with the most recent guidelines. It is not clear why this difference exists, but Grasemann *et al*.[13] have shown that it is partly associated with the NO synthase 1 genotype in healthy females; factors related to hormone production are less plausible, as Morris *et al*.[14] have shown that there is no temporal relationship between the measurements of NO production and urinary sex steroid conjugates during the menstrual cycle, thus suggesting that estrogens do not modulate FE_NO _concentrations.

We speculate that a further possible reason is the difference in airway surface area and calibre [15,16]. The same flow rate in airways of different calibres may differently dilute NO, which moves by means of gaseous diffusion into a smaller lumen (i.e. in females), thus leading to a lower NO concentration. Brooks *et al*.[17] have demonstrated that there is no within-gender correlation between tracheal size and body size or maximal expiratory flows, thus suggesting that the differences in the airway sizes of men and women are true gender-related difference and not simply due to differences in lung or body size. This hypothesis is in line with the findings of Buchvald *et al*. [7] showing no difference in FE_NO _levels between boys and girl of the same age, but a significant and positive relationship between FE_NO _and age (which leads to a progressive increase in airway surface) in both sexes; if the hypothesis is confirmed, it could be concluded that the low FE_NO _levels in women may simply be an artefact due to the use of a constant exhaled flow rate rather than a real reduction in NO airway production. In this regard, Nguyen *et al*. [18] have recently shown that the measurement of both FE_NO _and nitrogen oxides (NO_X_) in exhaled breath condensate is more indicative of airway NO production than FE_NO _alone; further studies should be carried out to verify whether there are any sexual differences in exhaled NOx.

Our data confirm that FE_NO _values are inversely related to the exhalation flow rate [19], and demonstrated that an exhaled flow of 50 mL/s was feasible in all our subjects, which is in line with the published guidelines [6].

We also measured FE_NO _levels at higher expiratory flows of 100 and 200 ml/s, because it has been suggested that extended exhaled NO measurements can distinguish alveolar and bronchial inflammation [20,21]. However, we found that there was a strong positive correlation (r = >0.7) between FE_NO _levels at different expiratory flows, and that it was not always possible to obtain reproducible expirations at higher flows in our healthy subjects. Further studies of large numbers of patients with proximal and distal airway inflammation should therefore be carried out in order to evaluate whether the FE_NO _measurements at different expiratory flow rates may lead to information that is as useful as that obtained at 50 ml/s.

The mean FE_NO _levels at different expiratory flows in our subjects are comparable with those previously reported by some authors in healthy non-smoking subjects[18,21], but slightly lower than those reported by others, particularly those observed at the expiratory flow rate of 50 ml/s [23]. In this regard, Borrill et *al*.[24] have recently compared the FE_NO _levels measured using three different commercially available analysers and found significant differences between them. This raises the important question of variability between NO analysers. This is an important point as Muller *et al*. [25] have recently shown that the main factors responsible for the different NO readings provided by different analysers are differences in calibration gases and procedures. Our study was not intended to compare the NO readings provided by different analysers, but we are confident of the reliability of our results because Borrill *et al*.[24] found that the most reproducible data was that obtained using the CLD88, probably because it has the lowest detection limit and fastest response time, and because it is CE MDD approved and totally compliant to the standard required by the ATS/ERS recommendations.

In conclusion, our study demonstrated that measuring FE_NO _measurement at an expiratory flow rate of 50 ml/s was feasible in a population of 204 healthy subjects aged 19–65 years, and indicates that different normal FE_NO _values should be defined for males and females.

## Abbreviations

ATS = American Thoracic Society

CE MDD= European Community Medical Device Directive

FE_NO _= fractional exhaled NO

ERS = European Respiratory Society

NO_X _= nitrogen oxides

Ppb = parts per billion

## Competing interests

The author(s) declare that they have no competing interest.

## Authors' contributions

OM: substantial contribution to study conception and design, sample collection, data acquisition, analysis and interpretation; involved in drafting the article.

GT: substantial contribution to study conception and design, data analysis and interpretation, critically reviewing the draft for important intellectual content.

MC: substantial contribution to study conception and design, sample collection; involved in drafting the articles.

LP: substantial contribution to study conception and design, data interpretation and statistical analysis; involved in drafting the article; final approval of the version to be published.

AM: substantial contribution to study conception and design, data interpretation and statistical analysis; involved in drafting the article; final approval of the version to be published.

CT: substantial contribution to study conception and design, data interpretation and statistical analysis; involved in drafting the article; final approval of the version to be published.

MM: substantial contribution to study conception and design, sample collection, critically reviewing the draft for important intellectual content.

**Figure 1:**
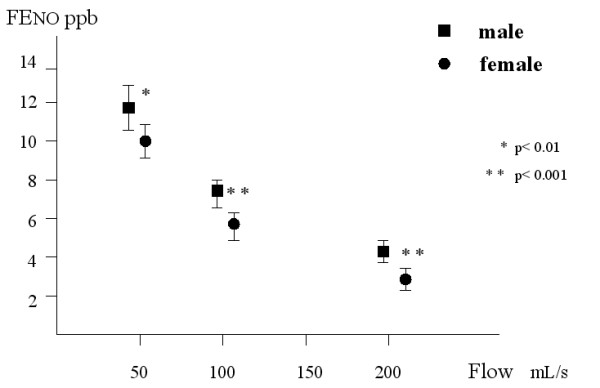
Fractional exhaled nitric oxide (FE_NO_) levels in men and women at three expiratory flows. Mean values and 95% confidence intervals.
